# Fiberoptic monitoring of central venous oxygen saturation (PediaSat) in small children undergoing cardiac surgery: continuous is not continuous

**DOI:** 10.12688/f1000research.3-23.v3

**Published:** 2014-06-13

**Authors:** Francesca G. Iodice, Zaccaria Ricci, Roberta Haiberger, Isabella Favia, Paola Cogo

**Affiliations:** 1 Department of Cardiology and Cardiac Surgery, Pediatric Cardiac Intensive Care Unit, Bambino Gesù Children’s Hospital, IRCCS, Rome, 00165, Italy

## Abstract

**Background:** Monitoring of superior vena cava saturation (ScvO _2_) has become routine in the management of pediatric patients undergoing cardiac surgery. The objective of our study was to evaluate the correlation between continuous ScvO _2_ by the application of a fiber-optic oximetry catheter (PediaSat) and intermittent ScvO _2_ by using standard blood gas measurements. These results were compared to those obtained by cerebral near infrared spectroscopy (cNIRS).

**Setting:** Tertiary pediatric cardiac intensive care unit (PCICU).

**Methods and main results:** A retrospective study was conducted in consecutive patients who were monitored with a 4.5 or 5.5 F PediaSat catheter into the right internal jugular vein. An *in vivo* calibration was performed once the patient was transferred to the PCICU and re-calibration took place every 24 hours thereafter. Each patient had a NIRS placed on the forehead. Saturations were collected every 4 hours until extubation. Ten patients with a median age of 2.2 (0.13-8.5) years and a weight of 12.4 (3.9-24) kg were enrolled. Median sampling time was 32 (19-44) hours: 64 pairs of PediaSat and ScVO2 saturations showed a poor correlation (r=0.62, 95% CI 44-75; p<0.0001) and Bland Altman analysis for repeated measures showed an average difference of 0.34 with a standard deviation of 7,9 and 95% limits of agreement from -15 to 16. Thirty-six pairs of cNIRS and ScVO2 saturations showed a fair correlation (r=0.79, 95% CI 0.60-0.89; p<0.0001) an average difference of -1.4 with a standard deviation of 6 and 95% limits of agreement from -13 to 10. Analysis of median percentage differences between PediaSat and ScvO2 saturation over time revealed that, although not statistically significant, the change in percentage saturation differences was clinically relevant after the 8th hour from calibration (from -100 to +100%).

**Conclusion:** PediaSat catheters showed unreliable performance in our cohort. It should be further investigated whether repeating calibrations every 8 hours may improve the accuracy of this system. CNIRS may provide similar results with a lower invasiveness.

## Introduction

Postoperative pediatric patients who have undergone cardiac surgery may benefit from venous saturation monitoring to assess oxygen delivery and as an indirect method of systemic perfusion ^1, 2^. A true mixed venous sample (SvO2) is drawn from the tip of the pulmonary artery catheter and includes all of the venous blood returning from the head and arms (via superior vena cava), the gut, the kidneys and lower extremities (via the inferior vena cava) and the heart (via the coronary sinus). In recent years, however, superior vena cava oxygen venous saturation (ScvO _2_) has replaced mixed venous saturation in many clinical settings and it is considered a reliable surrogate of SvO _2_^3^. In neonates and pediatric patients, the placement of a pulmonary artery catheter is not routine and may be problematic. In these patients ScvO _2_ monitoring plays an important therapeutic role ^4^. ScvO _2_ monitoring can be done intermittently (by the “traditional” co-oximetry method with serial blood withdrawals) or by reflectance oximetry through a fiber-optic catheter ^4, 5^. A new dedicated multilumen (PediaSat; Edwards Lifesciences, Irvine, CA, USA) central venous catheter (CVC) with incorporated fiber-optic technology for continuous oxygen saturation monitoring has been designed for use in neonates and pediatric patients. If correctly placed with the distal tip in the superior vena cava, this catheter is able to provide a reliable on-line measurement of the ScvO _2_^4, 6^. There is evidence that continuous monitoring of ScvO2 may have beneficial effects in the resuscitation of septic patients and after complex congenital heart disease ^7, 8^. According to manufacturer’s recommendations, after the first calibration the PediaSat catheter should provide consistent information for the following 24 hours. After this a new calibration is recommended in order to correct for potential drift from the true value ^9^.

Near-infrared spectroscopy (NIRS) is a noninvasive technique that measures continuous regional tissue oxygenation and is routinely used during pediatric cardiac surgery. NIRS measures the percentage oxygenated hemoglobin level in tissue beds. It is commonly used to determine cerebral tissue oxygen saturation (cerebral NIRS or cNIRS) and renal somatic tissue oxygen saturation (renal NIRS). cNIRS has been proposed to estimate adequacy of oxygen delivery due to a significant correlation existing between ScvO2 and cerebral NIRS ^10– 13^.

The objective of our study was to evaluate the correlation between continuous ScvO2 and intermittent SvO2 by using standard blood gas measurements during specific time points in postoperative pediatric cardiac patients. In particular we aimed to verify when calibration of a continuous ScvO2 catheter should be repeated in order to optimize the detection of potentially significant differences between continuous ScvO2 and intermittent ScvO2. Furthermore we also evaluated the correlation between cNIRS and intermittent SvO2.

## Materials and methods

A retrospective observational study was conducted in a tertiary pediatric cardiac intensive care unit from January 2013 until May 2013. Children undergoing elective cardiac surgery for congenital heart disease, and who had a PediaSat catheter placed (the indication was given by the attending anesthesiologist) at the Bambino Gesù Children’s Hospital, Rome, Italy, were enrolled in the study. Inclusion criteria were: 1) patient had been scheduled for elective cardiac surgery with cardiopulmonary bypass; 2) the patient was clinically indicated for central venous catheter placement with a size of 4.5 to 5.5 F; 3) the patient’s age was within the selected range: newborn >38 weeks gestation to child <10 years old; 4) the patient’s weight was >3.0 kg. Exclusion criteria were: 1) emergency operation; 2) need for or decision to position a femoral central venous catheter.

The INVOS 5100C Cerebral Oximeter (Somanetics, Troy, MI, USA) was used in all patients undergoing pediatric cardiac procedures with cardiopulmonary bypass according to the institutional protocol.

After induction of anesthesia, tracheal intubation, and radial or femoral artery cannulation, a central venous PediaSat catheter was inserted into the superior vena cava through the right internal jugular vein. The catheter was inserted using ultrasound guidance. Correct positioning was confirmed via transesophageal echocardiography and by a post-operative chest x-ray. The size and length of the catheters were decided on the basis of the patients’ weight. After catheter insertion an *in vivo* calibration was performed and repeated once the patient was transferred to the cardiac intensive care unit (CICU). The catheter was re-calibrated every 24 hours thereafter. Following induction of anesthesia, a NIRS sensor was placed on the patient’s forehead after adequate scrubbing of the skin.

### Data collection

Data were retrieved from the institutional database and missing data were acquired from patients’ clinical charts. Demographics, surgical procedure, cardiac bypass time and cross clamp time were recorded. In order to obtain ScvO2, blood was withdrawn from the distal port of the PediaSat catheter. Blood gases were analyzed in heparinized 1 ml syringes, within 60 seconds from withdrawal, with the GEM4000 blood gas analyzer (Brennan & Company, Dublin, Ireland). For each ScvO2 value, a PediaSat and cNIRS saturation was collected. According to the institutional protocol, patients’ oxygen saturations are reported into clinical chart every 4 hours until extubation (the first value being reported 1 hour after calibration). After patient extubation, data collection was terminated.

### Statistical analysis

A Spearman test was chosen for correlation estimation, and a Bland Altman analysis for repeated measures was used to verify bias and agreement of correlated variables. Results are expressed as median (interquartile range). Wilcoxon signed rank test was used for paired group comparison. One-way analysis of variance (Kruskal-Wallis non parametric test) was applied in order to evaluate difference of saturations as measured by the PediaSat and ScvO2 over time. Agreement between the two methods for tracking changes in SvO2 was quantified using polar plots: acceptable calibration was defined as an angular mean bias of less than ±5° and the percentage of data points lying within radial limits of ±30° from the polar axis was assessed ^14^. A P value <0.05 was considered significant. Statistical analysis was performed with the GRAPHPAD PRISM 5.0 software package (GraphPad Software, San Diego, CA, USA).

Institutional review board (“Comitato Etico per la Sperimentazione Clinica”) approved the study and waived the need for parental informed consent due to the retrospective nature of the study.

## Results

**Figure 1.**
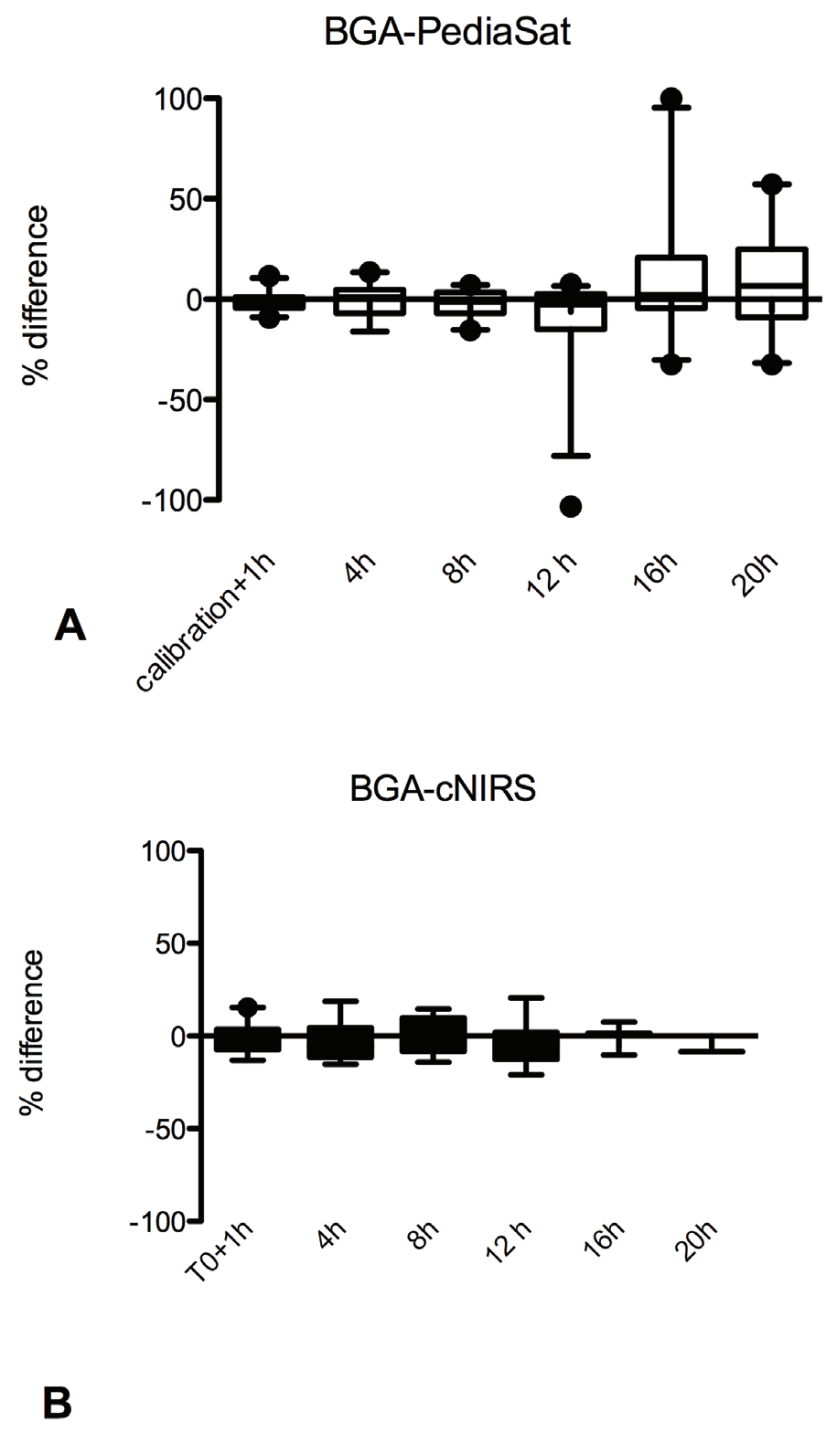
Saturation differences over time. Percentage difference between ScvO2 values obtained by blood gas analysis (BGA) and PediaSat I ( **A**), and BGA and cerebral near infrared spectroscopy (cNIRS) ( **B**), expressed over time.

Ten patients with a median age of 2.2 (0.13–8.5) years and a weight of 12.4 (3.9–24) kg were enrolled. Median mechanical ventilation duration was 36 hours (12–48). Cardiologic diagnoses and surgical procedures with cardiopulmonary bypass (CPB) details are reported in Table 1. Median sampling time was 32 (19–44) hours: at 48 hours all patients were extubated and the data collection never lasted longer than this. Sixty-four pairs of PediaSat and ScvO2 saturations were available: 5 missing pairs are acknowledged (on the clinical chart, once was ScvO _2_ value alone reported and 4 times no value was reported). Median PediaSat venous saturation was 71 (64–81)% whereas the median ScvO2 value was 74 (64–82) (p=0.347). Correlation between these two methods was poor (r=0.62, 95% CI 44–75; p<0.0001). Bland Altman analysis for repeated measures revealed a bias of 0.34 with a standard deviation of 7.9 and 95% limits of agreement from -15 to 16. Thirty-six pairs of cNIRS and ScVO _2_ saturations were also available for analysis: median cNIRS venous saturation was 74 (62–78)% whereas median ScvO2 values were 71 (62–78) (p=0.150). Correlation between these two methods was fair (r=0.79, 95% CI 0.60–0.89; p<0.0001). Bland Altman analysis for repeated measures showed an average difference of -1.4 with a standard deviation of 6 and 95% limits of agreement from -13 to 10. Analysis of median percentage differences between PediaSat and ScvO2 saturation over time revealed that there was not a significant modification over the different timeframes (p=0.28) ( Figure 1A). However, although not statistically significant, the change of percentage saturation differences was clinically relevant especially after the 8 ^th^ hour after calibration when errors from -100% to +100% were noted both as under and overestimation by the PediaSat method ( Table 2). Similar results were found when percentage cNIRS-ScvO2 differences were evaluated (p=0.86) ( Figure 1B) although the error never exceeded 20% at any time point and cNIRS showed a slight tendency to systematic underestimation of true values ( Table 2). Trending ability of PediaSat as assessed by a Polar plot showed a mean angular deviation from the polar axis of 90°, with 50% of the data points lying outside the radial limits of ±30° from the polar axis. Trending ability of cNIRS assessed by Polar plot showed a mean angular deviation from the polar axis of 42° with 30% of the data points lying outside the radial limits of ±30° from the polar axis.

**Table 1.** Demographic data of enrolled patients. BSA: body surface area. LOMV: length of mechanical ventilation. CPB: cardiopulmonary bypass. Xclamp: cross clamp.

Number of patients=10								
	Weight (Kg)	Height (cm)	BSA	Age (years)	LOMV (days)	CPB (min)	Xclamp (min)	Sampling time (hours)
Minimum	3,100	50,00	0,2119	0,03288	1,000	54,00	64,00	16,00
25% Percentile	3,950	52,50	0,2435	0,1295	1,750	89,00	79,00	19,00
Median	12,40	82,50	0,5364	2,242	2,000	149,0	114,0	32,00
75% Percentile	24,25	123,3	0,8932	8,481	3,000	185,5	130,0	44,00
Maximum	30,00	133,0	1,050	11,48	3,000	295,0	195,0	44,00
Diagnosis	Mitral regurgitation	Pulmonary atresia with ventricular septal defect	2 × Transposition of the great arteries	Aortic arch interruption	2 × Truncus arteriosus	Tetralogy of Fallot	Mitral stenosis and subaortic stenosis	Univentricular heart with aortic arch hypoplasia
Surgery	correction	correction	correction	correction	correction	correction	correction	Damus Kaye Stansel procedure

**Table 2.** Median and range percentage differences between ScvO2 reference values (achieved by catheter withdrawn blood gas analysis) and PediaSat/cerebral Near Infrared Spectroscopy (cNIRS).

PediaSat						
Time points	Calibration + 1h	4 hours	8 hours	12 hours	16 hours	20 hours
Minimum	-9,231	-16,13	-15,38	-102,3	-32,35	-32,39
25% Percentile	-4,181	-6,918	-6,935	-14,89	-4,433	-8,943
Median	-1,809	1,086	-1,220	-2,564	2,103	6,542
75% Percentile	1,022	4,727	3,374	2,599	20,70	24,91
Maximum	11,59	13,46	7,046	7,500	100,0	57,32
**cNIRS**						
**Time points**	**0 hour + 1h**	**4 hours**	**8 hours**	**12 hours**	**16 hours**	**20 hours**
Minimum	-13,04	-15,38	-14,06	-20,91	-10,29	-8,451
25% Percentile	-7,432	-11,52	-8,326	-12,62	-10,29	-8,451
Median	-1,299	-5,857	-4,435	-5,852	1,587	-8,451
75% Percentile	3,603	4,224	9,798	2,096	7,500	-8,451
Maximum	15,38	18,57	14,54	20,55	7,500	-8,451

Post- operative clinical data of children enrolled for comparison of ScvO2% with PediaSat and with cerebral NIRS values IJV: internal jugular vein; SVC: superior vena cava; LOMV: length of mechanical ventilation; TIME0: data collection starting time point; TIME 4: data collection after 4 hours from start (and so on); CVP: central venous pressure; LAP: left atrial pressure; SpO2 (%): systemic oxygen saturation; NIRSc (%): cerebral near infrared spectroscopy saturation; EGASVO2 (%): superior vena cava oxygen saturation (blood gas analysis); PEDIASVO2 (%): superior vena cava oxygen saturation (PediaSat catheter); NA: not available . Click here for additional data file. Copyright: © 2014 Iodice FG et al. 2014 Data associated with the article are available under the terms of the Creative Commons Zero "No rights reserved" data waiver (CC0 1.0 Public domain dedication).

## Discussion

Evaluation of oxygen delivery is optimized by measurement of continuous superior vena cava oxygen saturation. This is due to the fact that unexpected/sudden low cardiac output events may occur in the timeframe between serial ScvO2 evaluations; of note, the trigger for occasional venous samplings (hypotension, need for increasing vasoactive drugs dose, etc.) may also be prompted untimely with respect to cardiac decompensation. The ideal device to perform this continuous monitoring should be accurate and minimally invasive, it should provide at least two saturation values per minute, it should be applicable to smaller patients and, finally, it should be cost-effective. To date, such a device is not yet available in routine clinical practices. The PediaSat catheter does certainly have some of these features and several initial reports seemed to provide encouraging results in clinical practice ^2, 4, 5^. Unfortunately, the application of PediaSat to our small cohort of patients below 10 kg of body weight provided unsatisfactory results: the PediaSat catheter provided unacceptable saturation differences, with respect to the reference ScvO2 values (-26 to 25%). Furthermore, such deviation from actual values did not show a systematic error on the device and the PediaSat did not display a consistent over- or underestimation of true ScvO2 values: hence adjustments did not seem possible in order to correct the measures. In terms of trend estimation, PediaSat provided fair results, although still far from being adequate in terms of reliable routine utilization. The reasons for its lack of accuracy are not completely clear, but they could be caused by imperfect functioning of the miniaturized technology. It is also possible that more frequent calibrations should be performed: in our cohort, precision of PediaSat started to decrease, in a clinically significant way, after the 8 ^th^ hour after calibration and this trend was similar after each calibration (as seen in the subgroup of patients who passed the 24 ^th^ monitoring hour and whose PediaSat catheter was therefore re-calibrated). On the other hand, cNIRS, whose application is favored due to its low invasiveness, showed similar results, if not slightly better, in terms of coupling with ScvO2 values, limits of agreement at Bland Altman analysis, drift from actual values over time and trending ability. It must be remarked, however, that cNIRS values may be significantly affected by ventilation, sedation, cardiac anatomy and temporal distance from the surgical procedure ^13^.

Our results conflict with some reports ^2, 4, 5^ and agree with others ^15^. However, this is one of the first studies evaluating PediaSat in routine practice, and not during a specifically designed study. Furthermore, we hypothesize that PediaSat catheter might improve its performance with the calibration repeated every 8 hours.

Our study is certainly limited by the small subgroup of patients that may have potentially caused a bias in the statistical analysis: as a matter of fact, correlation and ANOVA should be calculated, in a larger population, for repeated measures (as done for Bland Altman). Finally, we acknowledge that the comparison between PediaSat and cNIRS monitor may be biased by the fact that cNIRS values were fewer in number and this may have randomly reduced the possibility for cNIRS errors. For these reasons we cannot definitely state which of the two monitoring techniques performs better.

In conclusion, 4.5 and 5.5 F PediaSat catheters showed unreliable performance during the early post operative course of children below 10 kg, who underwent cardiac surgery with CPB. It should be further investigated if repeating calibrations every 8 hours instead of every 24 hours may improve the accuracy of this system. At the moment cNIRS provides similar results with a lower invasiveness.

## Data availability

The data referenced by this article are under copyright with the following copyright statement: Copyright: © 2014 Iodice FG et al.

Data associated with the article are available under the terms of the Creative Commons Zero "No rights reserved" data waiver (CC0 1.0 Public domain dedication). 

*figshare*: Post-operative clinical data of children enrolled for comparison of ScvO2% with PediaSat and with cerebral NIRS values. doi: http://dx.doi.org/10.6084/m9.figshare.900915 ^16^
