# Supplementary material for: Fiberoptic monitoring of central venous oxygen saturation (PediaSat) in small children undergoing cardiac surgery: continuous is not continuous
Source: F1000Res. 2014 Jun 13;3:23. Originally published 2014 Jan 23. [Version 3] doi: 10.12688/f1000research.3-23.v3 (PMC3983937; doi:10.12688/f1000research.3-23.v3)
Supplement: Peer review history

# Peer review: Referee response for version 3

**DOI:** 10.5256/f1000research.4853.r5117

**Published:** 2014-06-18

**Authors:** Mirela Bojan

**Affiliations:** 1 Department of Anesthesia and Critical Care, Necker Sick Children's Hospital, Paris, France

I have no further comment.

I have read this submission. I believe that I have an appropriate level of expertise to confirm that it is of an acceptable scientific standard.

# Peer review: Referee response for version 2

**DOI:** 10.5256/f1000research.4216.r4505

**Published:** 2014-05-22

**Authors:** Mirela Bojan

**Affiliations:** 1 Department of Anesthesia and Critical Care, Necker Sick Children's Hospital, Paris, France

I thank the authors for considering all of the remarks, it was a pleasure to read the revised manuscript which, I believe, is improved. Albeit I believe this is a valuable report, I am unsure that the figures highlight the message. My (last) suggestion is use a trellis chart: if time is on the abscissa axis, and the three individual measurements (BGA, Pediasat, cNIRS) on the ordinate axis, than this should result in 10 boxes, each box showing all measurements per patient along time. I believe it will become obvious to the reader that the Pediasat measurement becomes unreliable after 8 hours, and requires calibration.

I have read this submission. I believe that I have an appropriate level of expertise to confirm that it is of an acceptable scientific standard.

# Peer review: Referee response for version 2

**DOI:** 10.5256/f1000research.4216.r3313

**Published:** 2014-05-09

**Authors:** Sabino Scolletta

**Affiliations:** 1 Department of Anesthesiology and Intensive Care, University of Siena, Siena, Italy

The authors have improved their manuscript. The statistical analysis is correct. The results are well presented and the discussion is focused.

I thank the authors for having followed my suggestions.

Sincerely,

Sabino Scolletta

I have read this submission. I believe that I have an appropriate level of expertise to confirm that it is of an acceptable scientific standard.

# Peer review: Referee response for version 1

**DOI:** 10.5256/f1000research.3529.r3867

**Published:** 2014-04-04

**Authors:** Mirela Bojan

**Affiliations:** 1 Department of Anesthesia and Critical Care, Necker Sick Children's Hospital, Paris, France

I have read with pleasure the article by Dr Ricci and colleagues. The topic is of high interest, since SvO _2_ measurements represent standard monitoring in the postoperative management of several pediatric cardiac procedures. Also, ScvO _2_ is nowadays an accepted surrogate measurement of SvO _2_. Significant differences have been reported between continuous fiberoptic measurements and intermittent measurements in adults with cardiac surgery ( Baulig *et al., *2008), requiring repeated calibration of the continuous measurement instrument. The findings here suggest a requirement for calibration every 8hrs, albeit the population is too small to allow for conclusions.

I believe there is a bias in the statistical analysis, since correlations have been estimated between repeated measurements – as a matter of fact, there is a temporal autocorrelation between the results of a same patient at different time points. However, this bias should result in overestimation of the relationship. The negative result concerning ScvO _2_-BGA correlation is somewhat “reinforced” by this bias. But one can question about the positive NIRS-BGA correlation. I understand that the population is too small to calculate correlations at each time point, or to analyse data using a mixed model for repeated measurement. Therefore, I suggest presenting the results in Figure 1 and Figure 3 separately for each patient, showing on the same graph the BGA, ScvO _2_ and NIRS measurements, and allow for visual assessment of the relationship in each patient at each time point. This should reassure the reader about the potential bias due to temporal autocorrelation. Since the correlation between ScvO _2_-BGA is negative, and since the authors do not demonstrate but only suggest a positive correlation between NIRS-BGA, this should be acceptable.

I have read this submission. I believe that I have an appropriate level of expertise to confirm that it is of an acceptable scientific standard.

# Response

**Published:** 2014-04-05

**Authors:** Zaccaria Ricci

**Affiliations:** Department of Pediatric Cardiosurgery, Bambino Gesù Hospital, Italy

We wish to thank Dr Bojan for her valued suggestions; these are in line with the comments of Sabino Scolletta. As recommended, we will change the figures as per improved visualization of data and leave the remaining results unchanged. As remarked by the reviewer, the new figures will reassure the reader, in order to cope for repeated measure analysis, although confirming the results presented in the text.

# Peer review: Referee response for version 1

**DOI:** 10.5256/f1000research.3529.r3896

**Published:** 2014-02-27

**Authors:** Sabino Scolletta

**Affiliations:** 1 Department of Anesthesiology and Intensive Care, University of Siena, Siena, Italy

In this article, Dr. Iodice and associates presented the results on the correlation between continuous and intermittent ScvO _2_ values, obtained with a fiber-optic oximetry catheter (PediaSat) and standard blood gas measurements, respectively. In addition, ScvO _2_ values were compared to those obtained by cerebral near infrared spectroscopy (cNIRS). The study was conducted on ten children undergoing cardiac surgery.

Although this is a retrospective observational study, it has been well described. Also, the article is clear, and the results are well presented. The discussion is very concise but highlights sufficiently some positive and negative aspects of the devices under study. Furthermore, it makes comparisons with other studies showing similar or opposite results on this field.  

My major criticism is about statistical analysis. Indeed, as the measurements of oxygen saturation have been performed at different times, the adjustment for the effects of repeated measurements in the Bland- Altman analysis had to be used. Conversely, the authors have used standard Bland-Altman and correlation analyses.

Despite the aforementioned criticism, I think the topic of this article is of interest. Indeed, monitoring tissue oxygenation in critically ill patients is crucial during cardiac surgery. In particular, children undergoing cardiac operations may benefit from assessing the adequacy of their oxygen delivery (DO _2_) with respect to their oxygen requirements. To this purpose, an indirect index of tissue perfusion is the mixed venous oxygen saturation (SvO _2_). However, to measure this parameter a pulmonary artery catheter (PAC) is required. Unfortunately, PAC is invasive and usually not practicable in children. Over the past years, the central venous oxygen saturation (ScvO _2_) has been used in many clinical settings as a surrogate of SvO _2_. Thus, ScvO _2_ can be measured continuously (fiber-optic catheter) or intermittently (blood withdrawals) and it is currently considered a possible alternative to SvO _2_. Besides, the measure of ScvO _2_ requires a less invasive technique, as for its measurement a central venous catheter is needed instead of a PAC.

In this study, the main result was that the fiber-optic catheter PediaSat showed unreliable performance. However, the authors had repeated measures from a small cohort of children with <10 kg of body weight. This may have biased their findings. Further studies are warranted, as conflicting results on this issue still remain in the current literature.

Of note, a key message from this investigation is that monitoring brain function with cNIRS is safe, continuous and easy to achieve in children undergoing cardiac surgery. In addition, this method has the main advantage of providing useful information on brain function, which can be considered a subtle marker of systemic hypoperfusion in case of low cerebral oxygen saturation. Monitoring cerebral function with cNIRS may avoid potential neurological complications, which are associated with increased morbidity and mortality, and poor quality of life.

I have read this submission. I believe that I have an appropriate level of expertise to confirm that it is of an acceptable scientific standard.

# Response

**Published:** 2014-03-01

**Authors:** Zaccaria Ricci

**Affiliations:** Department of Pediatric Cardiosurgery, Bambino Gesù Hospital, Italy

We are thankful to the reviewer for the precious suggestions and kind comments. We agree that the topic of assessing the adequacy of children's oxygen delivery (DO _2_) with respect to their oxygen requirements is crucial in pediatric intensive care and we are convinced that this topic has not been addressed effectively, so far. Indeed he is right: Bland Altman analysis for repeated measures should have been performed for our data analysis. This will be certainly modified in the revised version of the manuscript. Of note we already performed the corrected analysis and did not found significantly different results. In light of this we think that the overall message of the study has not been affected by our methodological mistake.
